# Antimicrobial prescribing in patients admitted through the emergency department of connolly hospital

**DOI:** 10.1186/1753-6561-9-S7-A15

**Published:** 2015-10-27

**Authors:** Lauren Messmer, Bernie Love, Eoghan O'Neill

**Affiliations:** 1 Royal College of Surgeons in Ireland, Dublin, Ireland; 2 Department of Pharmacy, Connolly Hospital Blanchardstown, Dublin, Ireland; 3 Department of Microbiology, Connolly Hospital Blanchardstown, Dublin, Ireland; 4 Department of Clinical Microbiology, Royal College of Surgeons in Ireland, Dublin, Ireland

## Background

Assessment of antibiotic prescribing is an important component of antimicrobial stewardship for the prevention of the misuse of antibiotics [1]. The aim of this study was to assess the prescribed antibiotics given to patients admitted through the Emergency Department (ED) of Connolly Hospital Blanchardstown (CHB) for compliance with the current CHB Guidelines for the use of Antibiotics [2].

## Methods

Antibiotics prescribed to patients admitted through the ED of CHB, over a three-week period in June - July 2014, were assessed for compliance with guidelines according to the documented indication for treatment. Data was obtained from patient charts and drug kardexes. Antibiotics prescribed by the ED and the subsequent antibiotics prescribed by the admitting team were recorded and evaluated for compliance with CHB antibiotic guidelines.

## Results

119 patients admitted through the ED in the study period were evaluated; of these, 24 patients (20%) were prescribed an antibiotic and included in the audit. 73% of the prescriptions given by the ED were compliant with the current CHB Guidelines for the use of Antibacterials. 88% of the subsequent prescriptions given by the admitting medical team were compliant with guidelines and 77% of the subsequent prescriptions given by the admitting surgical team were complaint with guidelines. Only 20% of community acquired lower respiratory tract infection (CA-LRTI) cases had a CURB-65 score calculated in correlation with antibiotic prescription. All non-compliance was due to choice of antibiotic.

## Conclusions

The majority of patients prescribed antibiotics admitted to CHB through the ED were commenced on antibiotics compliant with CHB guidelines with appropriate documentation and dose. There is, however, improvement required to raise this level of compliance and the results from this audit will form the basis for further prescriber education.
